# Antimicrobial Properties of Amniotic and Chorionic Membranes: A Comparative Study of Two Human Fetal Sacs

**Published:** 2017

**Authors:** Majid Zare-Bidaki, Sajad Sadrinia, Soheila Erfani, Ehsan Afkar, Nahid Ghanbarzade

**Affiliations:** 1- Infectious Diseases Research Center, Faculty of Paramedical Sciences, Birjand University of Medical Sciences, Birjand, Iran; 2- Faculty of Dentistry, Birjand University of Medical Sciences, Birjand, Iran; 3- Faculty of Medicine, Birjand University of Medical Sciences, Birjand, Iran; 4- Deputy of Research and Technology, Birjand University of Medical Sciences, Birjand, Iran

**Keywords:** Amniotic membrane, Antibacterial effect, Chorionic membrane

## Abstract

**Background:**

There is evidence of antibacterial properties of human chorioamniotic layer. However, the distinctive contribution of its individual parts, amniotic and chorionic membranes, to these effects is still unknown. The aim of present study was comparison of the antibacterial effects between amniotic and chorionic membranes.

**Methods:**

Chorioamniotic layer was removed from placenta belonging to 43 healthy mothers whose infants were delivered by caesarean section. Their amniotic and chorionic fetal tissues were manually peeled in sterile conditions. The antibacterial effects of all membrane samples were evaluated on 8 standard strains of bacterial collection using disk diffusion method on bacteriologic media. Results of bacterial growth inhibition in the presence of amniotic or chorionic membranes were measured and recorded as median±IQR. For data analysis and statistical comparison of samples, Kruskal-Wallis and Mann-Whitney U-test were applied using SPSS (v. 18).

**Results:**

Amniotic and chorionic membranes significantly showed different level of growth inhibitory effects on 8 bacterial strains including seven pathogens: *E. coli*, *Bacillus cereus*, *Klebsiella pneumonia*, *Streptococcus pyogenes*, *Pseudomonas aeruginosa*, *Staphylococcus aureus*, *Shigella flexneri* and one probiotic: *Lactobacillus plantarum* (p=0.018 and p<0.001, respectively). The number of bacterial growth inhibition zones around chorionic membranes was more than of what found around amniotic membranes.

**Conclusion:**

The superiority of antibacterial effects of the chorionic membrane compared with the amniotic membrane can represent the key role of maternal part in placenta in protecting the fetus against possible infections. The antimicrobial effect of amniotic and chorionic membranes is significantly different on various bacteria.

## Introduction

Although preterm birth can have many different causes, there is growing evidence that infection is a leading cause of preterm labors. Bacteria get into the amniotic cavity during pregnancy in various ways, such as crossing the placenta and hematogenous spread, as well as through vaginal colonization and they cause infection (1–3). Subclinical amniotic fluid infections may lead to premature deliveries and preterm labors (4). The fact that such infections are rare during pregnancy and fetus remains in a sterile environment until birth even when the potentially normal flora and dangerous bacteria have been colonized in vaginal area, indicates the factors preventing infectious organisms from growth in uterus and gestational sac.

The role of local defense mechanisms and factors, such as immunoglobulins and cytokines in preventing infant infection has been demonstrated (5). One of the factors playing an important natural role in disinfection of fetus or prevention of infection is an amniotic fluid, in which the fetus is floating. The other factor is chorionic and amniotic membranes surrounding this fluid.

The chorioamniotic mesodermal separation is indeed a two-layer, thin, semi-transparent, yet sturdy and high tensile strength membrane. The inner layer is known as amniotic membrane and is adjacent to the amniotic fluid. The outer layer which is called chorionic membrane is next to the uterus and is considered as a maternal part of the placenta. Amniotic and chorionic membranes are strongly attached to each other (6).

Although several studies including our previous study have reported the presence of antimicrobial properties in the chorioamniotic membrane (7–10), the search in international scientific data banks showed no cases of quantitative comparative study between the antibacterial properties of amniotic and chorionic membranes. Therefore, the present study aimed to evaluate and compare the antibacterial properties of the two amniotic and chorionic fetal membranes *in vitro*.

## Methods

### Study design and sampling:

The present study was an experimental study conducted in 2015. Chorioamniotic membranes of placenta belonging to 43 healthy mothers, whose infants had been born by Caesarean section, were sampled. C-section was selected due to the high risk of contamination of fetal membranes with maternal vaginal and intestinal flora during vaginal delivery. The mothers were selected randomly. Detailed examination of mothers was performed by a physician and relevant questionnaires were completed by the researcher. Although the placenta is a discardable tissue, the written consent of the mothers was the first condition for their entry into the study. Other inclusion criteria were ages between 19 (included) and 39 (included) years, completion of normal pregnancy, lack of tobacco and opioid addiction, lack of any infectious blood-transmitted diseases, no history of risky sexual behaviors and non-use of antibiotics within one month before sampling. The various steps of the research were conducted after approval of the Ethics Committee of Birjand University of Medical Sciences.

**Figure 1.**
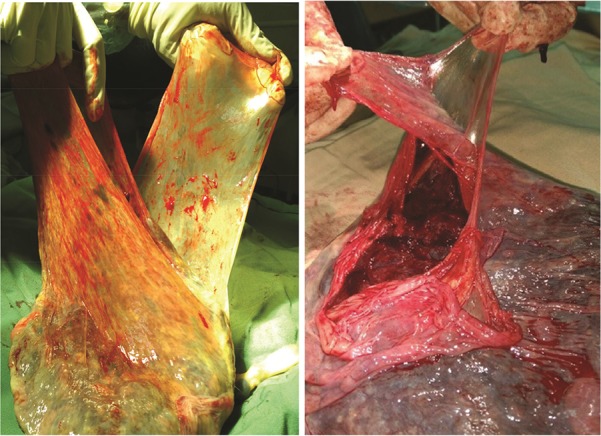
Peeling chorionic and amniotic membranes from each other in a placenta

Fetal chorioamniotic tissues of each placenta removed during c-section were manually peeled in sterile conditions (Figure 1). The peeled membranes were washed immediately and separately using sterile phosphate buffered saline solution (PBS) so that debris, blood and possibly remnants of amniotic fluid were washed away (8). The tissue samples were transferred to the laboratory at an interval of half an hour and at 4°*C*. Then, at least four 1×1 *cm* sized pieces were rapidly removed from each peeled amniotic and chorionic membranes using scalpel in a Class II biological safety cabinet. The resulting tissue pieces were kept in sterile PBS solution for a maximum of two hours at 4°*C* until the time for transition to bacteriologic media.

### Studied bacteria:

The tissue samples were changed to suspension at first. Antimicrobial effects of all fetal tissue samples were tested on eight standard bacterial strains including seven pathogens: *E. coli* (ATCC25922), *Bacillus cereus* (ATCC11778), *Klebsiella pneumoniae* (ATCC700603), *Streptococcus pyogenes* (ATCC19615), *Pseudomonas aeruginosa* (ATCC27883), *Staphylococcus aureus* (AT CC29213), *Shigella flexneri* (ATCC12022) and one probiotic: *Lactobacillus plantarum* (PTCC1745). All the strains were obtained in lyophilized stocks from Microbial Collection Department of Pasteur Institute of Iran.

### Cultivation of bacteria:

Bacterial recovery was carried out by inoculating lyophilized bacteria in nutrient broth medium (Merck, Germany) and incubating them for 24 *hr* at 37°*C*. The disk diffusion method was used to test the antibacterial effects of amniotic and chorionic membranes individually. Initially, 100 *μl* of each bacterial suspension equal to the McFarland 0.5 standard (1.5X10^8^ *CFU/ml*) was prepared in normal saline. Then, the bacterial suspensions were cultured in plastic petri dishes (80 *mm* DIC) containing Mueller Hinton Agar medium (Merck, Germany) using spread plate technique (8, 11). Exceptionally, the blood agar medium (Merck, Germany) was used to culture *Streptococcus pyogenes*. In the next step, the fragmented amniotic and chorionic membranes of each tissue sample were placed on the cultured petri dishes, so that their possible antibacterial components spread on the medium. For each bacterial strain, minimum of 4 pieces of amniotic membrane and 4 pieces of chorionic membrane of each placenta were used. Each of these quaternary sets was called a tissue group. Umbilical cord and a collection of antibiotic discs including erythromycin, ciprofloxacin, cefixime, ceftriaxone, cephalexin, amikacin, imipenem were used as negative and positive controls, respectively. All procedures were performed in accordance with Clinical and Laboratory Standards Institute (CLSI) guidelines (12).

In all culture cases, the plates were incubated at 37°*C* for 24 *hr* and finally the zone of inhibition (ZOI) of each bacterial strain around individual fragments of amniotic and chorionic membranes was measured using a caliper and recorded (13). In examining the culture results, only cases were recorded as growth inhibition, in which growth inhibition zone was a minimum of 1 *mm* around at least two out of four fragmented membranes of each tissue group.

In each plate, on which growth inhibition zone was observed, a swab was taken from the agar surface where a fetal membrane was laid as well as the marginal inhibition zone. The swabs were re-cultured and incubated for 24 *hr* at 37°*C* in the absence of any fetal membrane. The results were recorded in order to evaluate the bacteriostatic or bactericidal activity of the growth inhibition.

### Data analysis:

Initially, the normality of the data was controlled by the One-Sample K-S (Kolmogorov-Smirnov) Test. Results of bacterial growth inhibition in the presence of amniotic or chorionic membranes were reported as median±IQR. Data analysis was performed by SPSS (v.18) using Kruskal-Wallis and Mann-Whitney U-test.

## Results

### Growth inhibition:

**Figure 2.**
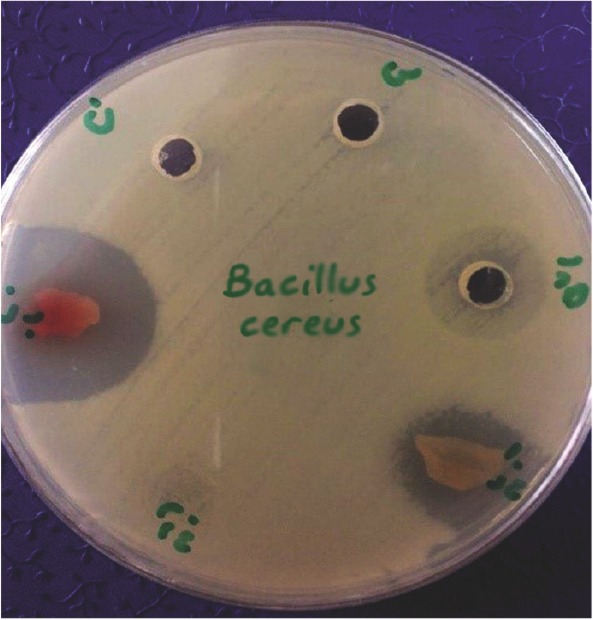
Formation of inhibitory zone of *Bacillus cereus* (ATCC11778) around amniotic tissue

**Figure 3.**
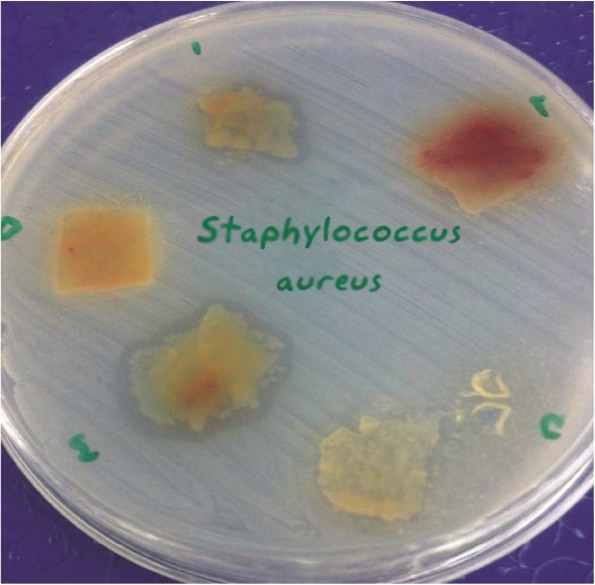
Formation of inhibitory zone of *Staphylococcus aureus* (ATCC29213) around amniotic tissue

The size of bacterial growth inhibition zones differed, depending on the type of bacteria, type of membrane (amnion or chorion) and the placenta sample. The inhibition zone size ranged between 0–12 *mm* (Figures 2 and 3).

**Figure 4.**
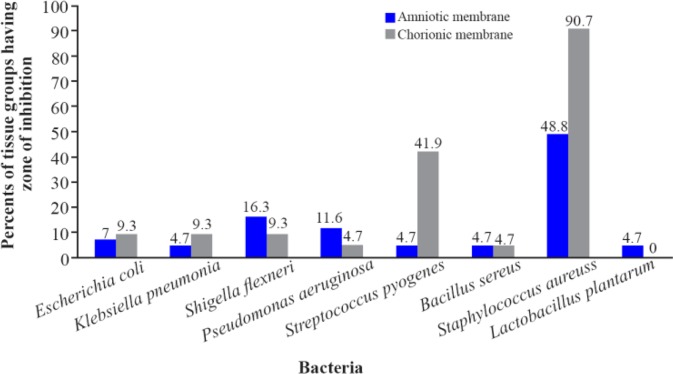
Comparing number of tissue groups having zone of inhibition with the total number of groups (%) in chorionic and amniotic membranes based on bacterial strain. The minimum and the maximum sizes of inhibitory zone were 1 mm and 12 *mm*, respectively

Growth inhibition zones around the amniotic membranes were observed in more than 70% of samples for all the bacteria other than *Staphylococcus aureus*. Growth inhibition zones around chorionic membranes were observed in more than 80% of samples for all the bacteria other than *Staphylococcus aureus* and *Streptococcus pyogenes* (Figure 4).

### Sizes of the inhibitory zones:

Each of the amniotic and chorionic membranes showed different sizes of inhibitory zone on the eight bacterial strains (Table 1). The number of growth inhibitions around chorionic membranes was significantly more than that of the amniotic membranes. These effects were mostly found in the chorionic membranes (Figure 1).

**Table 1.** The median values of growth inhibition zones around the amniotic and chorionic membranes

**Bacteria**	**Chorionic membrane** **Median±IQR**	**Amniotic membrane** **Median±IQR**	**p-value**
***Escherichia coli* (ATCC25922)**	8**±**7.25	3**±**1	0.212
***Klebsiella pneumonia* (ATCC700603)**	11.5**±**10.5	5**±**0.0	0.06
***Shigella flexneri* (ATCC12022)**	5.75**±**13	5**±**2	0.015
***Pseudomonas aeruginosa* (ATCC27883)**	3.5**±**16.5	5**±**3	0.053
***Streptococcus pyogenes* (ATCC19615)**	2**±**9.5	0.0**±**3.0	0.025
***Bacillus cereus* (ATCC11778)**	0.0**±**10.0	0.0**±**6.0	0.083
***Staphylococcus aureus* (ATCC29213)**	5**±**12	2**±**2	0.0001
***Lactobacilli plantarum* (PTCC1745)**	0.0**±**0.0	0.0**±**3.0	0.0001
**Statistical test (Level of significance)**	X^2^=24.41, p<0.001	X^2^=15.35, p=0.018	

Using Kruskal-Wallis test, the inhibition zones around the amniotic membranes were significantly different among the bacteria (p=0.018). Post-hoc analysis following a Mann-Whitney U-test showed that the inhibition zone for *Bacillus cereus* and *Klebsiella pneumoniae* was significantly greater than the one found for *Staphylococcus aureus* (p<0.01).

Performing similar analysis, the inhibition zone around chorionic membranes was significantly different among the bacteria (p=0.001). The inhibition zone for *Staphylococcus aureus* was significantly more than the one found for *E. coli* (p = 0.012) and *Streptococcus pyogenes* (p<0.001). Also the inhibition zone for *Pseudomonas aeruginosa* was more than the one found for *Streptococcus pyogenes* (p<0.05).

*Lactobacilli plantarum* revealed the minimum size of inhibitory zone around both amniotic and chorionic membranes. This strain significantly did not show zone of inhibition around any of the chorionic membrane samples (p<0.001).

No positive culture was found on plates inoculated by the swabs taken from agar surfaces where amniotic or chorionic membranes were laid.

## Discussion

The findings of the present study revealed that amniotic and chorionic membranes independently provide growth inhibitory effects on a varied and different range of bacteria. It also disclosed that the antimicrobial effects of fetal membranes are significantly stronger on some certain bacteria. Growth inhibition of *E. coli* and *Bacillus cereus* was found nearly at a similar level around both types of fetal membranes, which in some part was in agreement with what Parthasarathy et al. observed (11). However, *Pseudomonas aeruginosa*, *Staphylococcus aureus* and *Shigella flexneri* were more intensively under inhibitory effects of chorionic membrane which was not observed by Parthasarathy et al. This disagreement could rise due to some different reasons. They did not report the number of their tissue samples and also did not conduct any statistical analysis which might be due the low number of their tissue samples. Moreover, they worked only on clinical pathogens which might have different effects in comparison with the standard bacterial strains. In concordance with our study, in Kjaegaard et al.’s work, the growth inhibition was more pronounced for some of bacteria than for others (8). Similar findings of ours were previously reported for amniotic membrane by Tehrani et al., whereas they did not study antibacterial effects of chorionic membrane (14).

The most important finding of the current study was the superiority observed in antibacterial effects of the chorionic membrane compared with the amniotic membrane. The only previous study in this field is a descriptive and non-quantitative study conducted by Kjaergaard et al. in 2001 (8). Although they demonstrated antibacterial effects in both amniotic and chorionic membranes, more severe yet limited antibacterial effects was reported for chorionic membrane which had been observed only in the liquid medium. Perhaps the small sample size in their study made it impossible to analyze their data quantitatively for comparing the antibacterial effects of the two fetal membranes on solid medium.

According to our findings, when the swabs taken from the agar surface below the fetal membrane as well as the marginal inhibition zone, were cultured on a new agar plate, no bacterial growth was observed. One might speculate that the antibacterial effects in both amniotic and chorionic membranes are bactericidal. In the present study, which is an example of an *in vitro* model to test the antibacterial effects of fetal membranes, the above theory was presumably proved.

The nature of antibacterial factors in amniotic and chorionic membranes and the quantitative or substantive differences that are likely to exist between these fetal membranes are disputed. Kjaergaard et al. demonstrated that for applying antimicrobial effects of fetal membranes, no direct contact between the bacteria and tissue is needed. They managed to apply this property even through a filter on bacteria (8). Also, Tehrani et al. demonstrated that there is no difference between mesenchymal and optimal levels of amniotic membrane in terms of antibacterial effects (14). Generally, these findings suggest that the antibacterial effects in the fetal membranes are not tissue-dependent.

The presence of some specific productions of immune system, such as IgA in the fetal membranes in the edge detached from the placenta has been proved (15). Even Berezoswski et al. have demonstrated the presence of considerable amounts of this immunoglobulin in chorioamniotic membranes of women with premature rupture of fetal membrane (16). However, it is rather implausible that IgA could be a major factor of bacterial growth inhibition around the fetal membranes of healthy and full-term pregnancies in non-inflammatory conditions. Some other antimicrobial compounds have been reported in the fetal membranes. Human beta-defensin is a large group of natural antibacterial proteins produced by a number of epithelial cells including the chorioamniotic membranes (17, 18). HBD3 compound is a dominant defensin in the amniotic epithelial. Defensin’s inhibitory effects, especially β3-defensin, have been shown against a number of pathogens (18, 19).

Elafin and SLPI (Secretory Leukocyte Protease Inhibitor) are other anti-bacterial agents, the presence of which in amnion tissue has been reported (5, 20). *In vitro* studies have shown that SLPI is excreted by both Decidua levels, including Paritalis and Basalis (20). Both Elafin and SLPI are among the peptides that have anti-protease and elastase inhibition activity and as components of the innate immune system protect surfaces in contact with contamination by controlling the inflammatory responses in the mucosal surface. It has been shown that Elafin is secreted by the endometrial epithelial cells and amniotic membrane (5, 21). Elafin’s inhibitory effects against *Staphylococcus aureus* and *Pseudomonas aeruginosa* have been previously reported (22).

In addition to anti-bacterial protein compounds, the presence of compounds such as lactoferrin and hyaluronic acid, both of which are known as anti-inflammatory and antimicrobial properties, has been reported in the amniotic membrane (23, 24). In addition to antimicrobial compounds known in fetal membranes, several antibacterial factors have been also reported in amniotic fluid. The role of antimicrobial agents, including transferrin, lysozyme, 7S immunoglobulin, globulin β1c/β1a, IgA and also some peptides, such as α-defensins (HNP1-3) and calprotectin has been demonstrated in amniotic fluid (25–27). However, it does not seem that after the withdrawal of amniotic fluid, these agents still remain at the fetal membranes surfaces and play a role in the appearance of its experimental antibacterial effects on the culture media. More importantly, in the present study, the fetal membranes were carefully washed in PBS solution before use, in order to ensure that the amniotic fluid does not stay on their surface.

One might argue that the superiority of antibacterial effects of the chorionic membrane compared with the amniotic membrane can represent more effective and key role in maternal part of placenta in protecting the fetus against possible infections.

Despite conclusive experimental evidence indicating the existence of antimicrobial factors in the fetal membranes, no investigation report of quantitative comparisons of the type and frequency of antimicrobial factors between chorionic and amniotic membranes was found in international databases. Further researches are needed to clarify the exact contribution of each antimicrobial compound found in amniotic and chorionic membranes in the emergence of antibacterial properties of these membranes *in vitro* as well as to obtain a quantitative understanding of antimicrobial activities in each fetal membrane.

In this study, some limitations were encountered. One of the limitations was disagreement of some mothers for testing their placenta which reduced the number of membrane samples in general. The other limitation was the elastic nature of fetal membranes which made it difficult to cut off the membrane samples into fragments exactly at the same size.

## Conclusion

This study aimed to compare the antibacterial properties of amniotic and chorionic membranes using placental tissue of 43 Iranian women in Birjand, South Khorasan Province. This study revealed that not only the antimicrobial effect of amniotic and chorionic membranes is significantly different on various bacteria, but also the antibacterial effect level of chorionic membrane is greater than amniotic one.
